# *In crystallo*-screening for discovery of human norovirus 3C-like protease inhibitors

**DOI:** 10.1016/j.yjsbx.2020.100031

**Published:** 2020-07-16

**Authors:** Jingxu Guo, Alice Douangamath, Weixiao Song, Alun R. Coker, A.W. Edith Chan, Steve P. Wood, Jonathan B. Cooper, Efrat Resnick, Nir London, Frank von Delft

**Affiliations:** a Division of Medicine, UCL, Gower Street, London WC1E 6BT, UK; b Diamond Light Source, Harwell Science and Innovation Campus, Didcot, Oxfordshire OX11 0DE, UK; c Department of Biological Sciences, Birkbeck, University of London, Malet Street, Bloomsbury, London WC1E 7HX, UK; d Department of Organic Chemistry, Weizmann Institute of Science, Rehovot 7610001, Israel; e Structural Genomics Consortium, University of Oxford, Roosevelt Drive, OX3 7DQ, UK; f Department of Biochemistry, University of Johannesburg, Auckland Park 2006, South Africa

**Keywords:** X-ray crystallography, Structural biology, Fragment screening, Drug discovery, 3C-like protease, Anti-virals

## Abstract

Outbreaks of human epidemic nonbacterial gastroenteritis are mainly caused by noroviruses. Viral replication requires a 3C-like cysteine protease (3CL^pro^) which processes the 200 kDa viral polyprotein into six functional proteins. The 3CL^pro^ has attracted much interest due to its potential as a target for antiviral drugs. A system for growing high-quality crystals of native Southampton norovirus 3CL^pro^ (SV3CP) has been established, allowing the ligand-free crystal structure to be determined to 1.3 Å in a tetrameric state. This also allowed crystal-based fragment screening to be performed with various compound libraries, ultimately to guide drug discovery for SV3CP. A total of 19 fragments were found to bind to the protease out of the 844 which were screened. Two of the hits were located at the active site of SV3CP and showed good inhibitory activity in kinetic assays. Another 5 were found at the enzyme’s putative RNA-binding site and a further 11 were located in the symmetric central cavity of the tetramer.

## Highlights

• Noroviruses responsible for 99% of viral foodborne illness.
• Norovirus 3C-like protease is excellent drug target.
• X-ray fragment-screening gave a total of 19 fragment hits.
• Two located at the active site and showed inhibitory activity.
• Five found at the enzyme’s putative RNA-binding site and eleven in the central cavity of the tetramer.

## Graphical abstract

**Figure:**
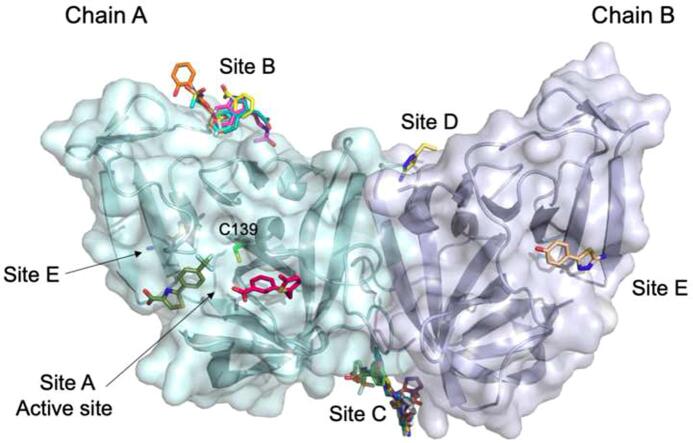

## 1 Introduction

Gastroenteritis accounts for the deaths of over 2000 children every day worldwide, making it the second leading cause of death for children under the age of 5, more than the combination of AIDS, malaria and measles (Liu et al., 2012). Whilst there are many other causes of gastroenteritis, including parasites, bacteria and viruses, human caliciviruses are recognised as the leading cause of gastroenteritis worldwide among people of all ages. The *Caliciviridae* family contains five genera known as norovirus, vesivirus, nebovirus, sapovirus and lagovirus (Clarke et al., 2012) with norovirus being the most common cause of disease in humans (Lambden et al., 1993).

Noroviruses account for more than 50% of gastroenteritis cases and at least 90% of nonbacterial acute gastroenteritis cases worldwide, as reported by the Centers for Disease Control and Prevention in the US (2011). Scallan et al. (2011) estimated that 99% of all viral foodborne illness incidents are caused by noroviruses which corresponds to 5.5 million per year in the US alone. From 2009 to 2013, around 62.5% of norovirus cases needed long-term care facilities in order to control the transmission (Vega et al., 2014). Statistics are generally similar in Europe (Baert et al., 2009, Phillips et al., 2010). Globally, it is estimated that noroviruses lead to a total of $4.2 billion in direct health system costs and $60.3 billion in social cost per year (Bartsch et al., 2016).

Clinical treatment and intervention is hampered by the lack of licensed vaccines or antivirals. Treatment with human immunoglobulin did show some benefit but did not result in clearance of the virus (Florescu et al., 2008). Whilst development of a vaccine has been hindered by the lack of small-animal models and cell culture systems, a number of norovirus vaccines are yielding promising results in clinical trials (e.g. Bernstein et al., 2015, Mateo et al., 2020). In general, norovirus vaccines are based on the use of virus-like particles formed by the main capsid protein VP1 (Lucero et al., 2018). A recent review of the development of norovirus antiviral agents and their targets is given by Netzler et al. (2019).

Noroviruses are genetically classified into 7 genogroups, GI - GVII, based on the amino acid sequence of the VP1 capsid protein and are further segregated into at least 40 genotypes (Vinjé, 2015). Noroviruses from groups GI (like Southampton virus) and GII infect humans, as do members of the GIV.1 subgroup. GII viruses are the most frequently detected (89%) while GII.4 are the major cause of norovirus outbreaks worldwide (Siebenga et al., 2009). Many noroviruses have been reported such as Norwalk virus (Jiang et al., 1993), Hawaii virus (Lew et al., 1994a), Snow Mountain virus (Lochridge and Hardy, 2003), Desert Shield virus (Lew et al., 1994b), Southampton virus (Clarke and Lambden, 1997) and Lordsdale virus (Lambden et al., 1993).

The norovirus genome consists of a single-stranded positive-sense RNA molecule of 7.5–7.7 kb in length and contains three open reading frames (ORFs) (Lambden et al., 1993), except for the murine norovirus which has a fourth alternative ORF (McFadden et al., 2011). ORF1 encodes a 200 kDa non-structural polyprotein which is co- and post-translationally cleaved into six or seven non-structural proteins by the viral 3C-like protease (NS6). The seven products of this proteolysis are, from N-terminus to C-terminus: p48 (NS1-2), an NTPase (NS3), a 3A-like protein (p22, NS4), a viral genome-linked protein (VPg, NS5), the 3C-like protease (3CL^pro^, NS6) and an RNA-dependent RNA polymerase (RdRp, NS7) (Blakeney et al., 2003). ORF2 and ORF3 encode the capsid protein VP1 and the minor structural protein VP2, respectively.

The 3C-like protease (3CL^pro^) was named because of its similarity to the picornavirus 3C protease. It is a cysteine protease which shows a typical chymotrypsin-like fold containing two domains: a *β*-barrel domain and a *β*-sheet domain separated by a groove where the active site is located (Bazan and Fletterick, 1988, Boniotti et al., 1994). The active site is characterised by a catalytic dyad (Cys139-His30) (Someya et al., 2002) or triad (Cys139-His30-Glu54) (Tiew et al., 2011) and shows a strong preference for a –D/E-F/Y-X-L-Q-G-P- (X can be H, Q, or E) sequence corresponding to the subsites S_5_-S_4_-S_3_-S_2_-S_1_-S_1_′-S_2_′ (Tiew et al., 2011). Studies have indicated that norovirus 3CL proteases have a preferential order of processing the polyprotein, for example, the Southampton virus 3CL^pro^ has a preference for cleavage at LQ-GP and LQ-GK, but it can also cleave at ME-GK, FE-AP and LE-GG (Hussey et al., 2011). Although several norovirus 3CL^pro^ structures have been determined (Hussey et al., 2011, Nakamura et al., 2005, Zeitler et al., 2006), the full structural basis of how these enzymes recognise these different sites is still unknown. The key role of norovirus 3CL^pro^ in the processing of the polyprotein and the absence of homologues in the human host make it an excellent target for antiviral drug discovery.

There is currently no clinically approved norovirus 3CL^pro^ inhibitor available but several compounds have been reported with strong inhibitory activity against 3CL proteases *in vitro*. These are usually peptidyl or macrocyclic compounds mimicking the substrate sequence whilst possessing a transition state analogue (Damalanka et al., 2017, Kankanamalage et al., 2015, Mandadapu et al., 2012). Examples include peptidyl aldehydes and *α*-ketoamides which showed strong inhibition of norovirus 3CL^pro^, and the 3C or 3C-like proteases in picornaviruses and coronaviruses in cell-based assays (Kim et al., 2012). The aldehydes and *α*-ketoamides act as warheads which form a reversible adduct with the catalytic residue Cys139 in the active site (Kim et al., 2012). These compounds are named as latent transition state (TS) inhibitors. TS mimics, such as *α*-hydroxyphosphonate, are converted to the aldehyde form either with or without catalytic action of the enzyme and form a tetrahedral adduct with the Cys139 residue (Kankanamalage et al., 2015). Hussey et al. (2011) first reported the X-ray structure of the Southampton norovirus 3CL^pro^ (SV3CP) with an inhibitor bound. This compound consisted of part of the most rapidly cleaved substrate sequence (EFQLQ) with a Michael acceptor moiety linked to the P_1_ residue Gln. This is attacked by Cys139 and a covalently bound complex is formed. Interestingly, the His30 sidechain is pushed away by the inhibitor, which disrupts the catalytic triad.

Screening by mass-spectrometry for covalent inhibitors of SV3CP has been described by us previously (Resnick et al., 2019). In this work we have crystallised the protease in its native form with an unperturbed catalytic triad and have conducted crystal-based fragment screening of 844 compounds with the aim of discovering novel inhibitory functional groups which have the potential to be developed as therapeutic agents, either on their own or through chemical coupling. A total of 19 compounds were found to bind to 3CL^pro^ in the crystals and two of them were located in the active site while another 5 were located at the enzyme’s putative RNA-binding site. A further 10 compounds were found to bind in the central cavity of this putative tetrameric form of the enzyme.

## 2 Methods

### 2.1 Crystallisation

Expression and purification of SV3CP was conducted using the method described by Hussey et al., (2011). Screening for crystallisation conditions for SV3CP was accomplished using the sitting-drop method at 21 °C with the screening kits: Structure Screen 1 & 2, JCSG-*plus*, PACT *premier*, MIDAS and Morpheus from Molecular Dimensions (Suffolk, UK). A TTP Labtech Mosquito crystal screening robot (TTP Labtech, Hertfordshire, UK) was used to dispense 400 nl of the protein, at concentrations of 5 mg/ml and 10 mg/ml, with 400 nl of the corresponding well solution into each drop. High quality crystals were obtained in 0.2 M ammonium citrate and 12% (v/v) PEG3350 after approximately one week, although crystals kept appearing over the next 2–3 months prior to screening.

### 2.2 Data collection, data processing and structure determination

Selected crystals were cryo-protected in 30% glycerol and mounted in loops before flash-cooling. X-ray data were collected at beamline I04-1 at Diamond Light Source (DLS, Didcot, England). Fine-sliced data were collected as guided by the strategy suggested by the program *EDNA* (Incardona et al., 2009). Data were processed automatically by the program x*ia*2 (Winter, 2010) at DLS, which revealed the space group to be *C*2, as shown in Table 1. Further analysis using *Phenix.xtriage* (Zwart et al., 2005) suggested that the data were of good quality. The solvent content of this crystal form was estimated to be 44.9% using *Matthews_coef* (Kantardjieff and Rupp, 2003).

**Table 1:** X-ray statistics for the native SV3CP structure and fragment complexes. Values in parentheses are for the high resolution shell. For the minority of structures where the overall fragment occupancy was either refined or is less than unity due to proximity with a symmetry axis, the fractional occupancy is shown following the mean fragment *B*-factor.

Fragment	Native	J01	J02	J03
PDB-ID	6t1q	6 t49	6t6w	6t2i
*a* (Å)	63.14	62.72	62.94	62.73
*b* (Å)	89.37	89.35	89.46	89.35
*c* (Å)	61.60	60.69	61.20	60.92
*β* (°)	96.50	96.57	96.89	96.95
Resolution (Å)	41.26–1.30 (1.33–1.30)	60.29–1.56 (1.60–1.56)	44.73–1.80 (1.85–1.80)	51.09–1.61 (1.65–1.61)
Completeness (%)	99.9 (99.9)	99.8 (99.8)	98.6 (99.4)	99.8 (99.7)
*R*_merge_ (%)	4.4 (139.6)	7.0 (78.0)	9.8 (66.8)	5.4 (75.6)
*R*_meas_ (%)	4.8 (152.5)	8.3 (99.1)	11.7 (79.3)	6.5 (96.7)
CC_½_ (%)	100.0 (55.3)	99.8 (45.4)	99.4 (64.7)	99.8 (62.1)
Mean *I*/*σ*(*I*)	13.6 (1.1)	7.2 (1.3)	8.9 (1.8)	8.3 (1.1)
Multiplicity	6.6 (6.2)	3.2 (2.6)	3.3 (3.4)	3.1 (2.5)
*N* observed	544,844 (38403)	149,418 (9035)	102,005 (7693)	135,174 (8082)
*N* unique	83,130 (6151)	47,120 (3473)	30,724 (2263)	43,021 (3176)
*R*-factor	15.08	15.34	14.40	15.40
Free *R*-factor	20.07	22.10	24.43	23.78
Test set size	4243	2236	1496	2154
R.m.s.d. bond lengths (Å)	0.015	0.013	0.011	0.013
R.m.s.d. bond angles (°)	1.88	1.84	1.80	1.87
Mean protein *B*-factor (Å^2^)	25.83	24.14	26.35	29.88
Mean fragment *B*-factor (Å^2^)	–	35.31	47.04, 0.86	25.55

Fragment	J04	J05	J06	J07
PDB-ID	6t4e	6t4s	6tbp	6t71
*a* (Å)	62.91	62.90	62.74	63.14
*b* (Å)	89.56	90.02	89.21	89.39
*c* (Å)	61.37	60.97	61.08	61.37
*β* (°)	96.64	97.06	96.82	96.73
Resolution (Å)	25.62–1.66 (1.70–1.66)	60.51–2.02 (2.07–2.02)	60.64–1.56 (1.60–1.56)	60.95–1.52 (1.56–1.52)
Completeness (%)	97.1 (95.9)	99.4 (98.9)	96.1 (82.9)	99.8 (99.6)
*R*_merge_ (%)	6.7 (84.8)	14.9 (113.0)	5.1 (56.1)	4.7 (53.6)
*R*_meas_ (%)	7.9 (105.3)	17.8 (134.8)	6.1 (71.1)	5.6 (67.6)
CC_½_ (%)	99.8 (42.5)	98.6 (50.9)	99.8 (67.4)	99.8 (78.8)
Mean *I*/*σ*(*I*)	12.4 (1.3)	5.3 (1.6)	9.0 (1.1)	9.6 (1.5)
Multiplicity	3.3 (2.9)	3.4 (3.3)	3.2 (2.6)	3.1 (2.5)
*N* observed	128,715 (7931)	73,672 (5243)	143,761 (7511)	162,852 (9643)
*N* unique	38,595 (2774)	21,956 (1583)	45,526 (2915)	51,832 (3801)
*R*-factor	13.71	21.36	13.74	13.85
Free *R*-factor	21.13	29.99	20.06	19.92
Test set size	1888	1052	2157	2517
R.m.s.d. bond lengths (Å)	0.012	0.009	0.012	0.012
R.m.s.d. bond angles (°)	1.89	1.75	1.76	1.83
Mean protein *B*-factor (Å^2^)	29.98	32.85	28.89	28.22
Mean fragment *B*-factor (Å^2^)	48.52	52.58	41.21	37.40

Fragment	J08	J09	J10	J11
PDB-ID	6t2x	6t5d	6t5r	6tc1
*a* (Å)	62.89	62.80	62.74	63.02
*b* (Å)	89.36	89.26	89.32	89.72
*c* (Å)	61.44	61.49	61.38	61.53
*β* (°)	96.73	96.67	96.80	96.67
Resolution (Å)	20.70–1.54 (1.58–1.54)	61.07–1.42 (1.46–1.42)	41.18–1.78 (1.83–1.78)	24.51–1.67 (1.71–1.67)
Completeness (%)	95.6 (85.5)	96.4 (86.8)	98.2 (99.5)	97.0 (95.7)
*R*_merge_ (%)	5.0 (88.9)	3.4 (67.2)	9.2 (81.2)	4.6 (79.6)
*R*_meas_ (%)	6.0 (112.6)	4.2 (90.8)	11.1 (97.3)	5.5 (99.5)
CC_½_ (%)	99.7 (50.4)	99.9 (50.2)	96.9 (59.3)	99.9 (46.9)
Mean *I*/*σ*(*I*)	12.7 (1.1)	15.0 (1.2)	11.7 (1.4)	15.2 (1.3)
Multiplicity	3.2 (2.6)	2.9 (2.1)	3.3 (3.2)	3.2 (2.8)
*N* observed	153,253 (8097)	179,818 (8428)	104,626 (7712)	123,219 (7726)
*N* unique	47,528 (3127)	60,967 (4032)	31,627 (2378)	38,160 (2789)
*R*-factor	13.72	13.12	14.13	12.83
Free *R*-factor	19.75	19.47	22.75	20.99
Test set size	2340	3026	1537	1849
R.m.s.d. bond lengths (Å)	0.014	0.015	0.011	0.012
R.m.s.d. bond angles (°)	1.91	2.04	1.82	1.81
Mean protein *B*-factor (Å^2^)	30.12	26.47	30.51	33.85
Mean fragment *B*-factor (Å^2^)	34.85	26.47, 0.50	44.42, 0.50	40.59

Fragment	J12	J13	J14	J15
PDB-ID	6tbo	6taw	6tgl	6t8t
*a* (Å)	62.74	62.93	62.88	63.09
*b* (Å)	89.46	89.03	89.20	89.51
*c* (Å)	61.15	61.35	61.18	61.07
*β* (°)	97.45	97.27	97.08	97.55
Resolution (Å)	31.11–2.07 (2.12–2.07)	51.11–1.41 (1.45–1.41)	25.10–1.99 (2.04–1.99)	25.07–1.68 (1.72–1.68)
Completeness (%)	98.1 (99.2)	96.0 (87.6)	98.9 (98.9)	99.4 (99.7)
*R*_merge_ (%)	17.8 (71.6)	4.1 (53.0)	10.3 (44.8)	6.4 (80.6)
*R*_meas_ (%)	21.4 (85.9)	4.9 (68.8)	12.3 (54.0)	7.6 (96.7)
CC_½_ (%)	97.8 (61.5)	99.9 (64.7)	99.3 (66.8)	99.8 (53.4)
Mean *I*/*σ*(*I*)	5.1 (1.6)	10.4 (1.1)	9.3 (2.8)	12.0 (1.5)
Multiplicity	3.2 (3.3)	3.0 (2.2)	3.3 (3.1)	3.4 (3.2)
*N* observed	65,116 (5021)	187,385 (9225)	74,353 (5270)	128,009 (9019)
*N* unique	20,052 (1524)	61,836 (4148)	22,739 (1682)	38,064 (2797)
*R*-factor	20.42	12.83	16.98	12.21
Free *R*-factor	28.99	17.79	23.71	19.39
Test set size	981	3151	1113	1911
R.m.s.d. bond lengths (Å)	0.008	0.014	0.008	0.013
R.m.s.d. bond angles (°)	1.60	1.89	1.54	1.78
Mean protein *B*-factor (Å^2^)	35.81	24.37	29.57	30.70
Mean fragment *B*-factor (Å^2^)	37.03	18.97	49.91	39.18

Fragment	J16	J17	J18	J19
PDB-ID	6t82	6tcf	6tal	6t8r
*a* (Å)	63.01	63.20	62.69	62.96
*b* (Å)	89.34	89.49	90.02	89.78
*c* (Å)	61.43	61.47	60.79	60.92
*β* (°)	97.08	96.93	96.23	96.42
Resolution (Å)	60.96–1.46 (1.50–1.46)	51.37–1.79 (1.84–1.79)	51.24–1.51 (1.55–1.51)	60.54–1.88 (1.93–1.88)
Completeness (%)	96.4 (86.0)	99.8 (99.7)	99.3 (98.4)	99.8 (99.8)
*R*_merge_ (%)	5.0 (62.7)	8.8 (55.1)	5.8 (68.0)	9.5 (66.5)
*R*_meas_ (%)	6.0 (80.0)	10.5 (65.3)	6.9 (86.9)	11.3 (79.6)
CC_½_ (%)	99.7 (73.9)	98.2 (80.4)	99.9 (64.1)	99.4 (68.4)
Mean *I*/*σ*(*I*)	8.8 (1.2)	6.5 (1.6)	9.3 (1.3)	5.9 (1.6)
Multiplicity	3.1 (2.4)	3.3 (3.4)	3.1 (2.5)	3.3 (3.3)
*N* observed	175,458 (8933)	106,031 (8062)	163,299 (9646)	89,757 (6547)
*N* unique	56,264 (3720)	31,951 (2374)	52,139 (3788)	27,348 (2005)
*R*-factor	13.75	14.52	14.35	13.70
Free *R*-factor	19.67	23.29	21.66	24.30
Test set size	2826	1559	2622	1339
R.m.s.d. bond lengths (Å)	0.015	0.011	0.013	0.010
R.m.s.d. bond angles (°)	1.90	1.70	1.80	1.65
Mean protein *B*-factor (Å^2^)	26.59	28.41	20.74	34.66
Mean fragment *B*-factor (Å^2^)	26.53, 0.50	40.47	23.78	56.50

The structure was determined by use of the program *Phaser MR* (McCoy et al., 2007) using the protein moiety of the published SV3CP-MAPI complex (PDB ID: 2iph) as a search model. Several rounds of manual rebuilding and correction were performed using *Coot* (Emsley and Cowtan, 2004) followed by restrained refinement using *Refmac*5 (Murshudov et al., 2011) and *Phenix.refine* (Afonine et al., 2012). Since the crystal diffracted to near atomic resolution, the temperature factors were refined anisotropically. Structure validation was performed with *MolProbity* (Chen et al., 2010). The statistics for data collection, data processing and refinement are shown in Table 1.

### 2.3 *In crystallo* fragment screening

#### 2.3.1 Crystal preparation

Crystals were prepared in Swissci 3-drop crystallisation plates (Hampton Research, CA, USA) in 200 nl droplets containing 100 nl of the protein (4 mg/ml) and 100 nl of well solution [0.2 M ammonium citrate, 12% (v/v) PEG3350]. Since all of the fragments were dissolved in 100% dimethyl sulfoxide (DMSO), crystal stability in this solvent was first tested in the range (v/v) of 0%, 10%, 20%, 30% and 40%, and on soaking time scales of 1 h, 3 h and overnight. In order to make the experiment more efficient, the crystals were also tested with and without additional cryo-protectant for data collection. It was found that these crystals could survive in 40% DMSO for many hours and additional cryo-protection was not required.

#### 2.3.2 Fragment soaking, crystal harvesting and data collection

The plates containing crystals were imaged using a Rock imager system (Formulatrix, USA). All the crystals were then ranked using the program *TeXRank* (Ng et al., 2014) and positional coordinates for the injection of the fragments were manually defined in the drop. Each fragment from the DSLP library (776 fragments) (Cox et al., 2016) and, due to time constraints, a subset of the Maybridge Ro3 core set (first 68 fragments) (Fisher Scientific UK Ltd, Loughborough, UK) was acoustically dispensed to the corresponding target position in droplets of 2.5 nl volume using a Labcyte Echo 550 liquid handler (Labcyte Inc, CA, USA) which gave an estimated final fragment concentration of 200 mM (Collins et al., 2017). Fragment soaking was conducted in batches to give an average soaking time of approximately 2.5 h prior to crystal mounting. Crystal harvesting was aided by the use of a crystallisation plate shifter (Oxford Lab Technologies, Oxford, UK). All the crystals were mounted in loops of about the same size as the crystals or slightly smaller to allow for automated, unattended data collection in which the X-ray beam was aimed at the centre of each loop. A total of 180° of data were collected for each crystal, taking approximately 60 s per crystal using DLS beamline I04-1.

#### 2.3.3 Fragment data processing, analysis and hit identification

The data produced were managed using *XChemExplorer* (Krojer et al., 2017) which gathered ligand information and data processing results and launched different software pipelines, such as *DIMPLE* (Wojdyr et al., 2013) for generating difference maps and *PanDDA* (Pearce et al., 2016) for further analysis and hit identification. *PanDDA* uses an average of several ground-state crystal structures to calculate a background density correction which reveals better electron density for weakly bound fragments. All the hits were checked visually by using the program *Pandda.inspect* in the *PanDDA* suite (Pearce et al., 2016). The hits were further refined using *Refmac*5 (Murshudov et al., 2011) followed by inspection using *Coot* (Emsley and Cowtan, 2004) for several rounds (Table 1). In most cases anisotropic *B*-factor refinement was undertaken and the fragment occupancy was fixed. Confirmatory omit maps for the ligands were generated using the program *Composite omit map* (Terwilliger et al., 2008) in the *PHENIX* program suite (Adams et al., 2010). Interactions between ligands and SV3CP were analysed using *LigPlot*^+^ (Wallace et al., 1995). Figures were prepared using programs *MarvinSketch* (ChemAxon, 2013), *PyMOL* (The *PyMOL* Molecular Graphic System, Schrödinger, LLC) and *CueMol* (Molecular Visualization Framework http://www.cuemol.org).

### 2.4 Activity assay

The protease (0.5 mg/ml final concentration) in a buffer containing 100 mM Tris, pH 8.5, and 5 mM β-mercaptoethanol was mixed with the fragment (dissolved in DMSO at concentrations of 0.027, 0.135, 0.27, 0.405 and 0.54 mM) for 20 min at RT. The solution was then mixed with the chromogenic substrate (Ac-EFQLQ-*para-*nitroaniline; Peptide Protein Research Ltd, Southampton, UK), which was dissolved in DMSO to give final concentrations of 0.4, 0.9, 1.4, 1.9, 2.5 and 3.0 mM, in a 1:1 ratio and the absorbance at 405 nm was measured at 20 s intervals over a 3 min period, using a Nanodrop ND1000 spectrophotometer. The *K*_i_ values were determined using *GraphPad Prism* (www.graphpad.com).

## 3 Results

### 3.1 Structure of native SV3CP

**Fig. 1:**
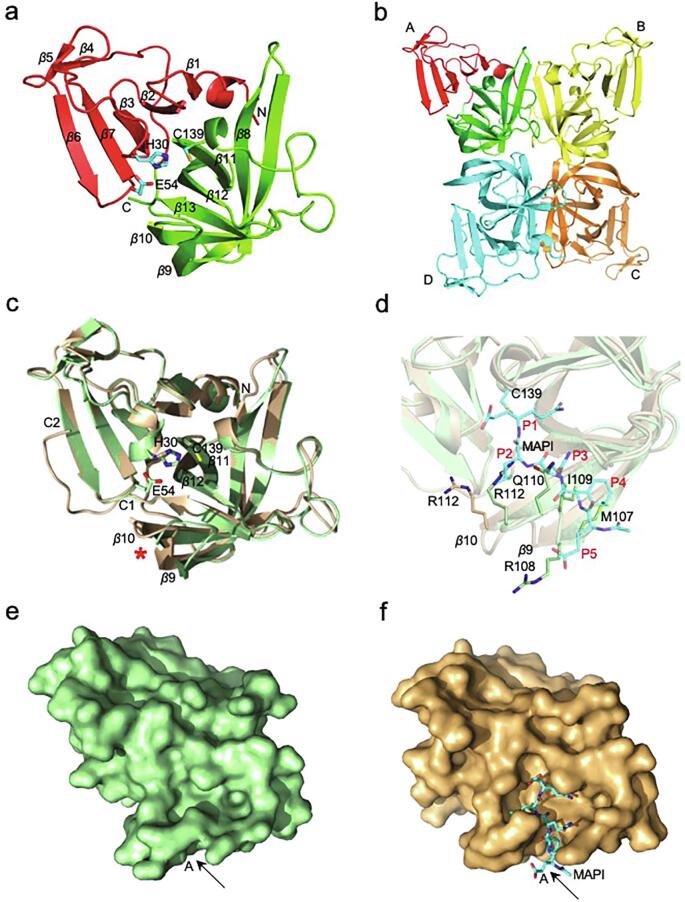
(a) The overall structure of SV3CP with the *β*-strands labelled. The protein is composed of an N-terminal domain (red) containing a twisted 7-stranded antiparallel *β*-sheet and a C-terminal domain (green) consisting of a 6-stranded *β*-barrel. The catalytic triad of Cys139, His30 and Glu54 is shown in stick representation. (b) The putative tetrameric form of SV3CP. The asymmetric unit is composed of a dimer formed by chains denoted A and B. Crystallographic symmetry generates another identical dimer formed by chains denoted C and D. Both the AB and CD dimers have significant buried surface area suggesting that the dimer is physiologically significant. However another stable interface is formed between chains A and D and between chains B and C, resulting a tetrameric ensemble which may also have physiological significance. The domains of chain A are coloured and oriented the same as in (a) whereas chains B, C and D are coloured yellow, cyan and ochre. (c) Superimposition of the protein moieties of ligand-free SV3CP (green) and the polypeptide inhibitor-bound structure (Hussey et al., 2011; RCSB ID: 2iph) (pale brown). The ligand-free SV3CP structure has a shorter C-terminal end labelled C1 whereas this part of the complex with the substrate-analogue inhibitor is labelled C2. The red asterisk indicates the *β*-hairpin (between strands *β*9 and *β*10) which undergoes substantial movement upon binding the inhibitor. (d) A close-up of the superposition showing the polypeptide Michael acceptor inhibitor (MAPI) coloured cyan and a number of residues in the *β*-hairpin, including Arg112 which moves drastically upon binding the inhibitor. (e) A surface representation of ligand-free SV3CP demonstrating the relatively closed state of the active site cleft (arrowed A). (f) A surface representation of the complex with MAPI in which the active site has opened appreciably to accommodate the polypeptide moiety of the inhibitor.

The structure of native SV3CP has been determined for the first time at the near-atomic resolution of 1.3 Å resolution (Fig. 1a) revealing a crystallographic tetramer (Fig. 1b). The monomers consist of an N-terminal and a C-terminal domain with the active site cleft located in between. As found in other noroviral 3CL^pro^ structures, the N-terminal domain contains an *α*-helix and a twisted 7-stranded antiparallel *β*-sheet forming an incomplete *β*-barrel (Anand et al., 2002, Birtley et al., 2005, Mosimann et al., 1997). The C-terminal domain is made up of 6 *β*-strands forming an antiparallel *β*-barrel and contains the catalytic cysteine residue (Cys139) which makes a catalytic triad with two residues from the N-terminal domain (His30 and Glu54; Fig. 1a). Interestingly, the *β*-hairpin formed by *β*9 and *β*10, which is involved in binding the N-terminal side of the substrate peptide, adopts an appreciably different conformation from that observed in an earlier inhibitor-complexed structure (Fig. 1c; Hussey et al., 2011). It is now clear that the backbone of this *β*-hairpin moves by over 7 Å to open up the active site cleft for substrate binding and movements of some of the side chain atoms exceed of 12 Å. Indeed, in the native enzyme, residues Met107 to Gln110 occupy very approximately the same positions as the P_5_ - P_3_ residues of the bound substrate analogue and the sidechain of Arg112 occupies the position of the P_2_ sidechain (Fig. 1d). In addition to the movement of *β*9 and *β*10, the *β*-hairpin formed by strands *β*11 and *β*12 also moves to some extent. These effects open up the active site, suggesting that a fairly marked conformational change occurs upon binding of substrate. The Michael acceptor inhibitor also pushes His30 away from the other members of the catalytic triad (Cys139 and Glu54, Fig. 1c).

The SV3CP enzyme has approximately 90% sequence identity with other GI noroviral 3C proteases and an identity of the order of 68% with the enzyme from the GII genotype. SV3CP has approximately 58% identity with the mouse norovirus enzyme. The monomer structures of these enzymes superpose with SV3CP with a Cα RMSD of typically 1.0 – 1.2 Å for virtually all of the amino acids in the chains. The structures differ most noticeably in the hairpin linking strands *β*9 and *β*10 which is close to the active site.

**Fig. 2:**
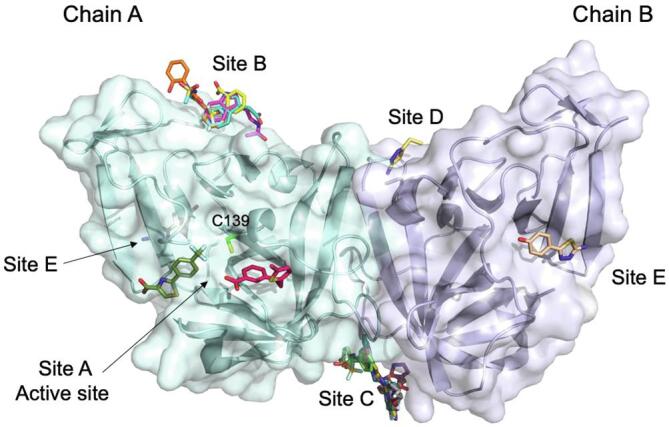
Binding sites of the fragment screening hits. All the ligands bind in five sites labelled as A – E which are shown on a surface representation of the dimer. Site A coincides with the active site of the protease and site B is a putative RNA binding site. Site C coincides with the centre of the tetramer.

In line with other noroviral 3C proteases which have been analysed by gel-filtration, it is highly likely that SV3CP forms dimers in solution or, at least, exists in a monomer – dimer equilibrium (Chang et al., 2012, Leen et al., 2012, Zeitler et al., 2006). Accordingly, a dimer is observed in the crystallographic asymmetric unit of SV3CP (Fig. 2, chains A and B). However, analysis with the *PDBePisa* website (Krissinel and Henrick, 2007) suggested a tetrameric form (Fig. 1b) might also be stable in solution. The interface area between the chains of the crystallographically observed dimers (formed by chains A and B) is 883.0 Å^2^. However, a neighbouring dimer in the crystal structure forms an interface of comparable buried surface area (692.3 Å^2^) between chains labelled A and D chains and likewise for chains labelled the B and C. This result indicates that higher order oligomers may possibly be formed by SV3CP dimers, such as the putative tetramer shown in Fig. 1b. Intriguingly, a number of other human GI and GII noroviral protease structures (Nakamura et al., 2005, RCSB ID: 1wqs; Muzzarelli et al., 2019, RCSB ID: 6b6i; Viskovska et al., 2019, RCSB ID: 6nir) form essentially the same tetrameric assembly in the crystals, as shown in Supplementary Fig. 1. The majority of the amino acids forming the tetramer contacts (Glu79, Met101, Ala103, Ala105, Ser106, Met107, Arg108, Met120, Leu121, Leu122, Thr161, Ser163, Asn165, Thr166) are either invariant or conservatively substituted. The same tetramer is also observed in mouse norovirus protease (Fernandes et al., 2015, RCSB ID: 4x2v) which has lower sequence identity with SV3CP (~58%) than do the other human GI or GII proteases (~91% and 68%, respectively). These findings, along with the ability of the tetramer cavity to bind small molecule fragments (see later), suggest that this tetrameric form may have functional significance for 3CL^pro^. Indeed, in the structure of the Minerva virus enzyme (RCSB ID: 6b6i) the tetrameric assembly allows the C-terminal ends of two monomers (equivalent to B and D) to extend into the active sites of adjacent monomers (C and A, respectively) across the dimer-dimer interface (Muzzarelli et al., 2019). Similar tail-interdigitating effects are observed in the structures of the protease from Houston virus (RCSB ID: 6nir; Viskovska et al., 2019) and mouse norovirus (RCSB ID: 4x2v; Fernandes et al., 2015). Given that localised replication centres are known to form within norovirus-infected cells (e.g. Thorne and Goodfellow, 2014), a high local concentration of 3CL^pro^ may allow the enzyme to tetramerise.

In the native SV3CP structure, no electron density is visible for the last 8 residues (ASEGETTL) at the C-terminal end of the protein. Since these residues are well-defined in the complex with a substrate analogue (Hussey et al., 2011), their absence in the native structure might be due to autolysis during storage or crystallisation of the uninhibited protease. In this region of the structure, there is a minor consensus sequence for SV3CP cleavage with the following amino acids VQ-AS corresponding to the P_2_-P_1_-P_1_′-P_2_′ positions (Hussey et al., 2011, Kankanamalage et al., 2015) suggesting that slow autolysis prior to crystal growth is possible. Mass spectrometric analysis of the purified protein yielded a molecular mass of 19,290 Da (Supplementary Fig. 2) confirming that the protease was indeed fully intact at the time of crystallisation. Therefore another possibility is that this region of the molecule is simply disordered in the new crystal form. However, it is not clear why this should be since this region of both monomers is not involved in crystal contacts in either crystal form.

### 3.2 Crystal-based fragment screening

Most crystals used in the non-covalent fragment screening experiment diffracted to resolutions ranging from 1.5 to 1.8 Å with good crystallographic statistics (Table 1). Fragment J12 is the worst in terms of resolution, diffracting to approximately 2.1 Å, although the electron density is still of good quality. Screening with the DSPL library and part of the Maybridge Ro3 library identified 19 ligands in total which bind in five different sites, as illustrated in Fig. 2. The majority of fragments have mean *B*-factors which are comparable with those of the protein moieties (Table 1). In only one case (J02) was the occupancy of the fragment refined, although for several others it was set to 0.5 due to the fragments residing on a 2-fold axis.

**Fig. 3:**
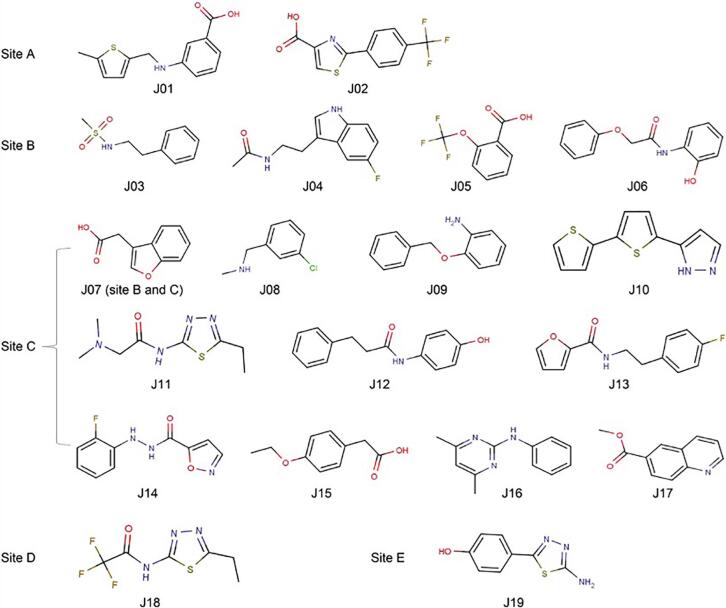
Molecular structures of the fragment screening hits.

Site A, the protease active site, is a long groove containing the catalytic Cys139 residue. Two fragments (J01 and J02) were found to bind here, each on different sides of the catalytic cysteine (Fig. 2). Five hits (J03-J07) were found to bind in the putative RNA binding site (site B) including one (J07) which also binds in another site, site C. Site C lies in a pocket between chains A and B and the symmetry related chains A’ and B’, with 11 hits being identified (J07-J17) here. Two other fragments were found at additional sites: D (J18) and E (J19). Molecular structures of the ligands J01-J19 are given in Fig. 3.

#### 3.2.1 Active site-binding fragments (site A)

**Fig. 4:**
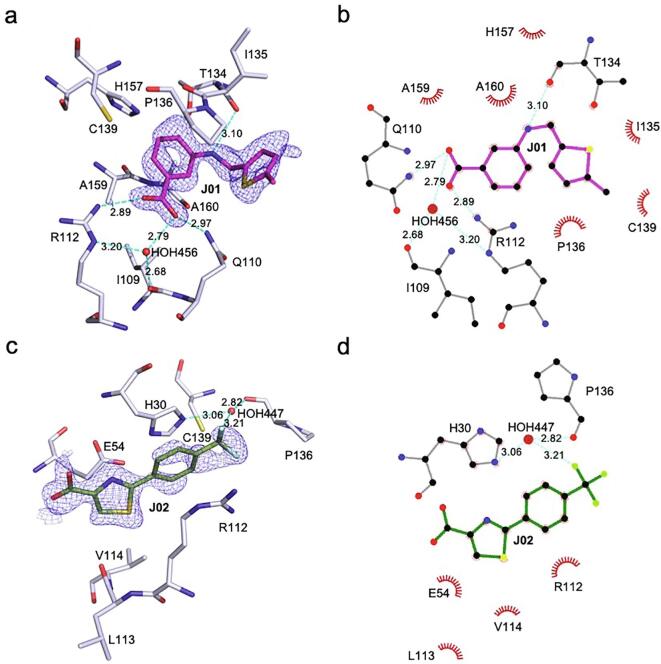
The two active site binding fragments (site A). J01 (magenta) and J02 (green) are shown in (a) and (c) with the omit maps (contoured at 1.0 RMSD) coloured blue. The interactions between SV3CP and these fragments are shown in (b) and (d), respectively. Hydrogen bonds are indicated by dashed lines in cyan with the corresponding donor–acceptor atom distances shown in Å. Hydrophobic interactions are indicated by red eyebrow-like icons.

Two non-covalently bound fragments were identified in the active site of the protease named as J01 and J02, as indicated by their omit maps (Fig. 4a and c). J01 binds in the S_1_ subsite where its carboxyl group is oriented towards S_2_ and S_3_. J01 forms several direct hydrogen bonds with the side chains of Gln110 and Arg112 and makes some additional hydrogen bonds mediated by a water molecule (Fig. 4a and b). These residues are at the tip of the functionally important *β*-hairpin (connecting strands *β*9 and *β*10) that is involved in substrate recognition and moves substantially upon binding of polypeptide substrate analogues (Fig. 1). However, in the presence of J01, the *β*-hairpin adopts the same conformation as the ligand-free SV3CP, suggesting that binding of this fragment does not alter its conformation. Since the carboxyl group of J01 appears to hold the *β*-hairpin loop (residues 109 to 112) in the closed conformation, this must help to prevent the enzyme from adopting the 'open' conformation that can accommodate the substrate. The ligand –NH group (N1) is also within hydrogen bonding distance of the main chain carbonyl group of Thr134. The benzoic acid moiety of J01 makes many hydrophobic interactions with the active site residues including Pro136, Cys139 and Ala160. In contrast, the 5-methyl-2-thienyl group forms fewer contacts with the enzyme than the aromatic group since it points away from the active site towards a large solvent channel.

J02 resides on the other side of the long active site, where it occupies the S_2_ subsite without forming any hydrogen bonds (Fig. 4c and d). Instead, the phenyl ring is sandwiched between the side chains of His30 of the catalytic triad and Arg112 from the *β*-hairpin loop by π - π stacking and cation - π interactions. Interestingly, the guanidinium group of Arg112 has moved from its position in the other fragment complex to accommodate J02. Several hydrophobic interactions are formed between this fragment and Glu54 from the catalytic triad and Val114, and a number of contacts are made with a symmetry-related molecule.

**Fig. 5:**
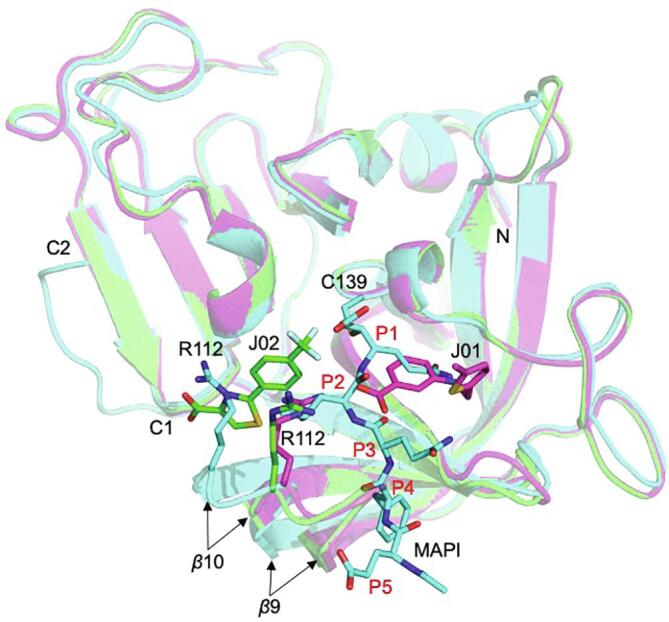
A superposition of the two active-site binding fragment structures on a covalently bound substrate analogue inhibitor. The complexes with J01 and J02 are coloured magenta and green, respectively, while the polypeptide Michael acceptor inhibitor structure (RCSB ID: 2iph; Hussey et al., 2011) is shown in cyan. The C-terminal end of ligand-free SV3CP is labelled C1 and the corresponding part of the polypeptide inhibitor complex is labelled C2. The *β*-hairpin loop connecting strands *β*9 and *β*10 moves significantly from its position in the native structure (which is very close to its position in the J01 and J02 complexes) upon binding the polypeptide inhibitor.

In kinetic assays both J01 and J02 showed inhibitory activity against SV3CP with *K*_i_ values of 0.37 mM and 0.34 mM, respectively. These values are typical of initial hits in crystallographic fragment-screening studies targetting catalytic- or allosteric-sites of enzymes (Bauman et al., 2013, Delbart et al., 2018, Zhang et al., 2019) suggesting that the binding modes we observe in 3CL^pro^ are highly relevant. Since J01 and J02 bind in the active site cleft and maintain the closed conformation of the hairpin, they are good candidates for developing further inhibitors and linking them into a new compound could also improve the bioactivity. A superposition of their binding modes on that of the covalently bound Michael acceptor inhibitor (Fig. 5) demonstrates how these two fragments occupy the S_1_ and S_2_ subsites, respectively. J02 does not overlap with the P_2_ residue of the polypeptide inhibitor as well as J01 and the P_1_ residue do, since it appears to lie somewhere between the spatially adjacent S_2_ and S_1_′ subsites.

#### 3.2.2 Fragments binding at the putative RNA binding site (site B)

In addition to the protease activity, studies on viral 3C proteases suggested that they or their larger precursors can bind specifically to the 5′-terminal nucleotides of the viral RNA (Leong et al., 1993, Nayak et al., 2006). The interaction occurs only on the plus strand which forms a ribonucleoprotein (RNP) complex that is necessary for the initiation of the plus strand synthesis (Andino et al., 1990). It has been shown that human noroviral RNA non-competitively inhibits the protease activity with an IC_50_ of in the µM range (Viswanathan et al., 2013). The RNA binding site has been studied by mutagenesis in other homologous 3C proteases, in which a key arginine residue was identified in the conserved sequence, KF/VRDI (F/V represents F or V) (Bergmann et al., 1997, Leong et al., 1993, Nayak et al., 2006). Structural comparison of SV3CP with HRV 3CL^pro^ (PDB ID: 5fx5; Kawatkar et al., 2016) and FMDV 3CL^pro^ (PDB ID: 2j92; Nayak et al., 2006) identified Arg65 as the equivalent residue in SV3CP, which is within a KIRPDL sequence that has similarity with the consensus. The R and D residues in this sequence interact by a salt-bridge that forms one side of the putative RNA binding site of SV3CP (site B) which is shown in Fig. 2 and, as for the FMDV and HRV proteases, it is a shallow groove. In addition, these sites are on the surface of the SV3CP tetramer and form deep channels with the neighbouring symmetry-related molecules in HRV, FMDV and Southampton virus 3CL^pro^. Inhibitors binding in the RNA binding site have the potential to inhibit noroviral replication and are therefore of interest as a separate class of drug.

**Fig. 6:**
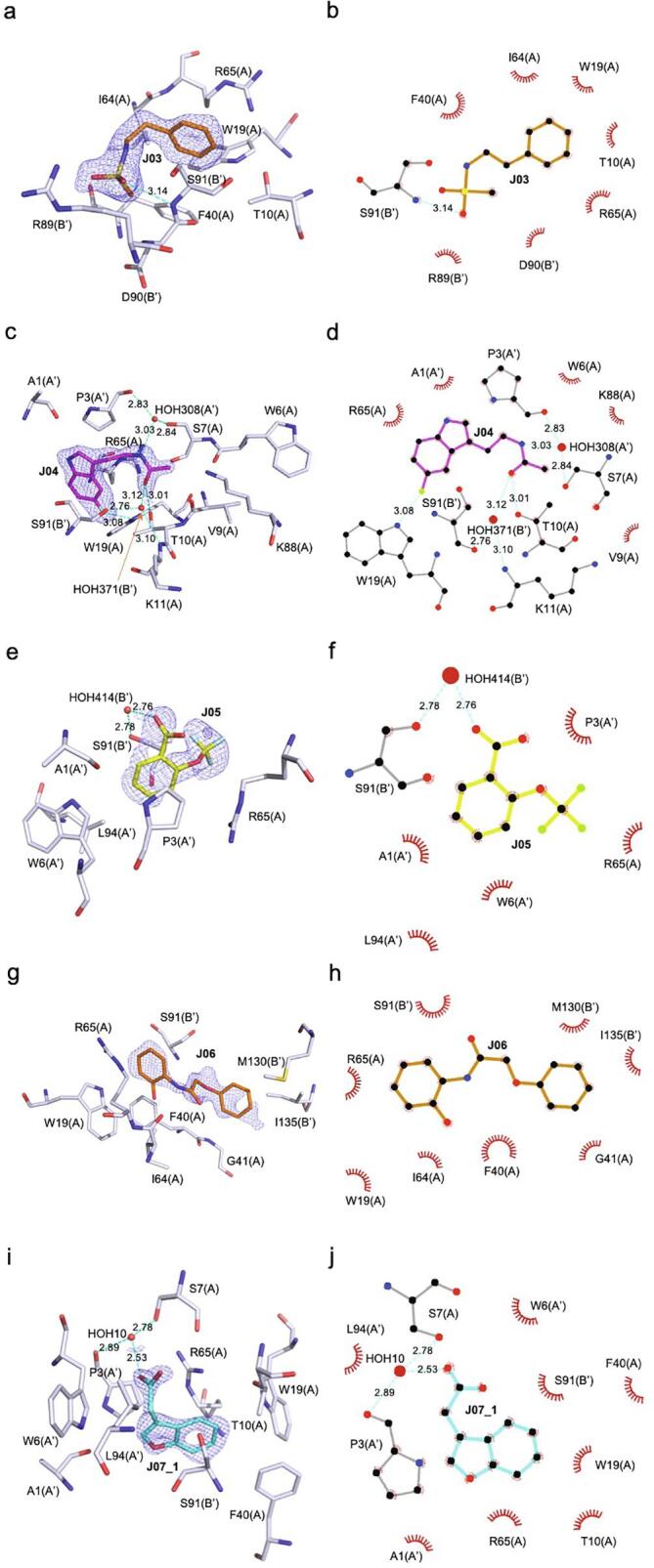
Interactions between the putative RNA binding site (site B) of the protease and fragments J03-J07. These are shown in 3D with the omit electron density contoured at 1.0 RMSD as (a, c, e, g, i) and in 2D with interacting residues shown in (b, d, f, h, j), respectively. Hydrogen bonds are indicated by dashed lines in cyan and hydrophobic interactions are indicated by red eyebrow-like icons. Protein chain identifiers are indicated by the letters A and B in brackets and those with a prime are from symmetry-related chains.

**Fig. 7:**
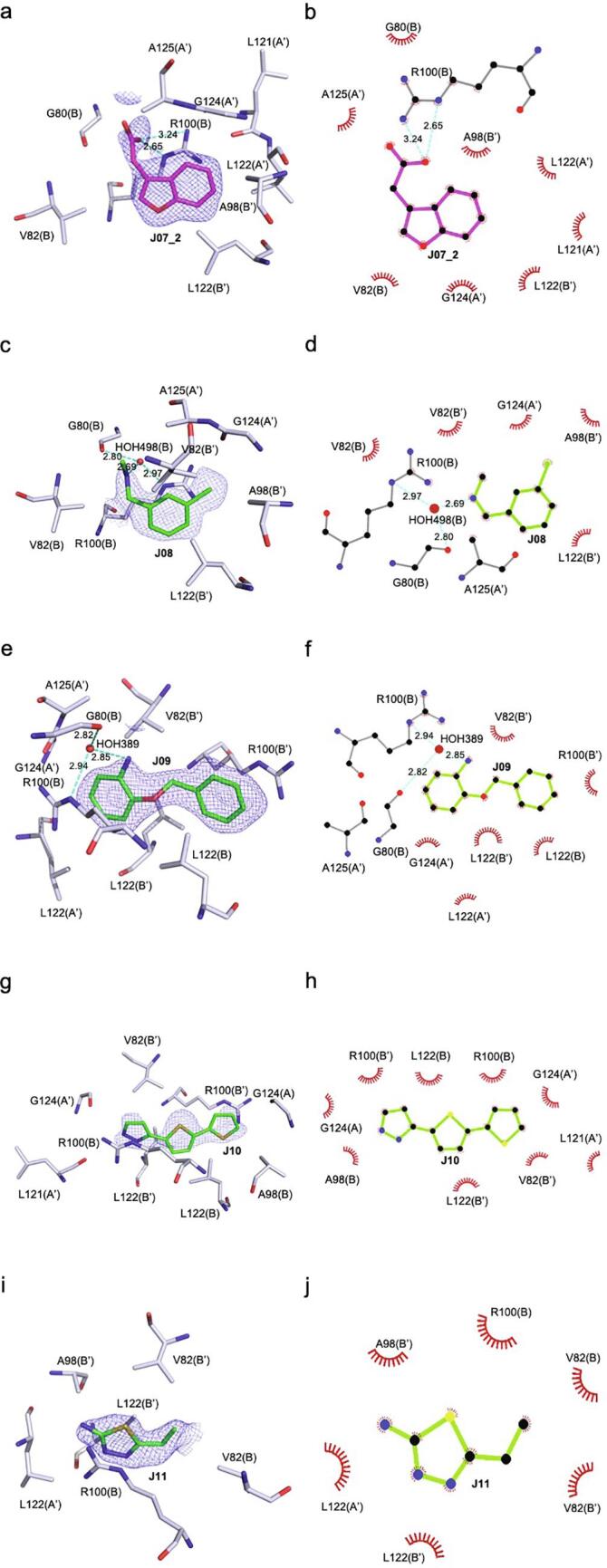

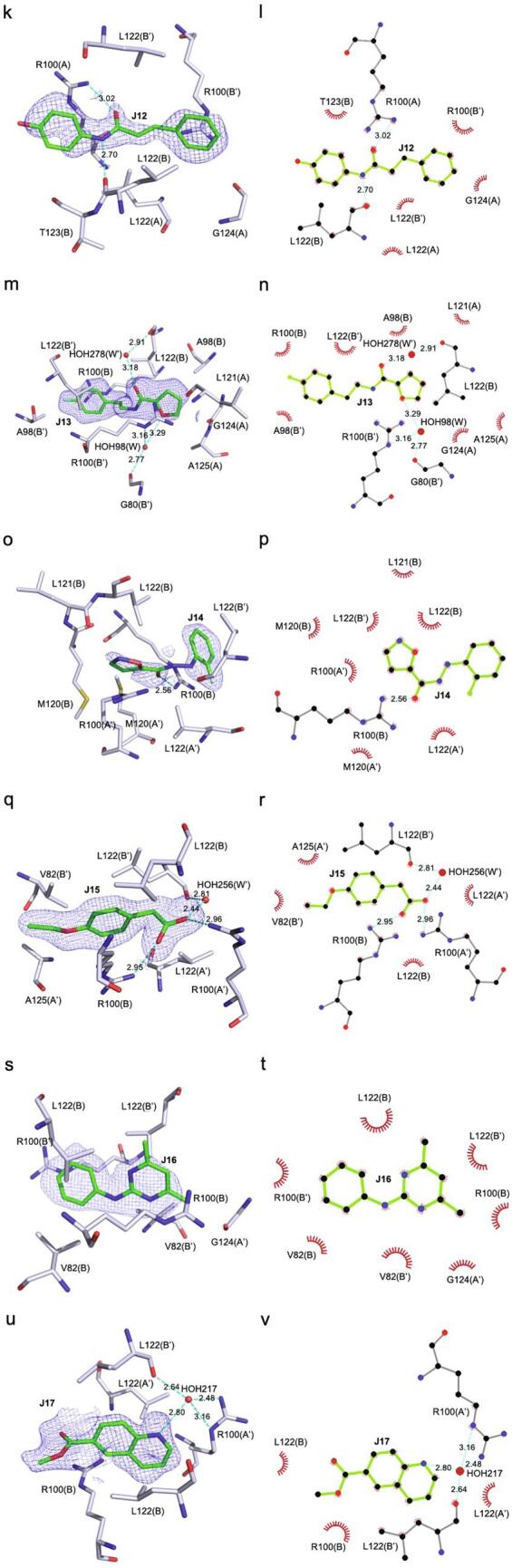
Interactions between SV3CP and fragments J07-J17 which bind in site C at the centre of the putative tetramer. These are shown in 3D with the omit electron density contoured at 1.0 RMSD as (a, c, e, g, i, k, m, o, q, s, u) and in 2D with interacting residues shown in (b, d, f, h, j, l, n, p, r, t, v), respectively. Hydrogen bonds are indicated by dashed lines in cyan and hydrophobic interactions are indicated by red eyebrow-like icons. Protein chain identifiers are indicated by the letters A and B in brackets and those with a prime are from symmetry-related chains.

Fragments J03-J06 were found to reside at this site and their contact residues are shown in Fig. 6. All the fragments form hydrophobic contacts with Arg65 and other residues in the KIRPDL sequence. While J03 (Fig. 6a and b) and J06 (Fig. 6g and h) are mainly involved in hydrophobic interactions, J04 (Fig. 6c and d) and J05 (Fig. 6e and f) also form many hydrogen bonds with the neighbouring residues, potentially making them stronger binders. The carbonyl group (O1) of J04 is involved in three hydrogen bonds formed, directly or mediated by a water molecule, with Thr10, Lys11 and Ser91 (although the latter residue is from a symmetry related molecule). The N1 atom forms two hydrogen bonds with Ser7 and Pro3 (also from the symmetry mate) with the participation of a water molecule. A hydrogen bond is also seen between the fluorine substituent in the indole ring of J04 and the NE1 atom on the side chain of Trp19. This residue is one of a number of quite solvent-exposed aromatic residues including phenylalanines 12, 25, 39 and 40 which form the putative RNA-binding site. J05 also forms water-mediated hydrogen bonds with Ser91 from the symmetry related molecule. Unlike the active site fragments which bind in different subsites of the substrate-binding channel, these four fragments bind in approximately the same position with their aromatic 'heads' overlapping to a large degree but their aliphatic 'tails' pointing away in different directions. Since binding of viral RNA inhibits the protease activity (Viswanathan et al., 2013), ligands binding at this site have the potential both to interfere both with RNA binding and with the protease activity. However, since this site is of the order of 20 Å from the catalytic centre the mechanism of protease inhibition is currently difficult to explain. Fragment J07 was found to bind in both the putative RNA binding site (B, Fig. 6i and j) and site C (Fig. 7a and b) in the centre of the putative tetramer.

#### 3.2.3 Fragments binding in the tetramer cavity (site C)

The finding that the native crystals of the enzyme are formed by a tetrameric assembly of monomers is suggestive of a physiological role for the tetramer. We were also intrigued to find that the majority of the fragments binding to the protease (J07 - J17, Fig. 7) were located in a cavity at the centre of the putative tetramer, site C. The site is characterised by the convergence of two-fold symmetry axes, both crystallographic and non-crystallographic, since the NCS two-fold relating the monomers in each dimer and the crystallographic two-fold relating both dimers in the tetramer meet at this point. The binding site is formed by four copies of the hydrophobic amino acids Leu122 and Val82 as well as Arg100 which are provided by all chains of the tetramer. These residues have a high level of sequence conservation. The sidechain of the arginine tends to form extensive stacking interactions with the aromatic moieties of the ligand. Since this site is formed at the convergence of 2-fold axes, two copies of each ligand are present at this site and sometimes the two symmetry-related copies of the fragment interact extensively with each other. Since the same tetrameric assembly is observed in other GI and GII norovirus proteases, this binding site may be a conserved feature of these enzymes. Given its ability to bind so many heteroaromatic fragments and the diverse functions which noroviral proteins and their precursors are known to have (e.g. Emmott et al., 2019), it is tempting to speculate that the tetramer cleft has a physiological role, perhaps even as a secondary substrate- or RNA-binding site.

#### 3.2.4 Other fragment binding sites (D and E)

**Fig. 8:**
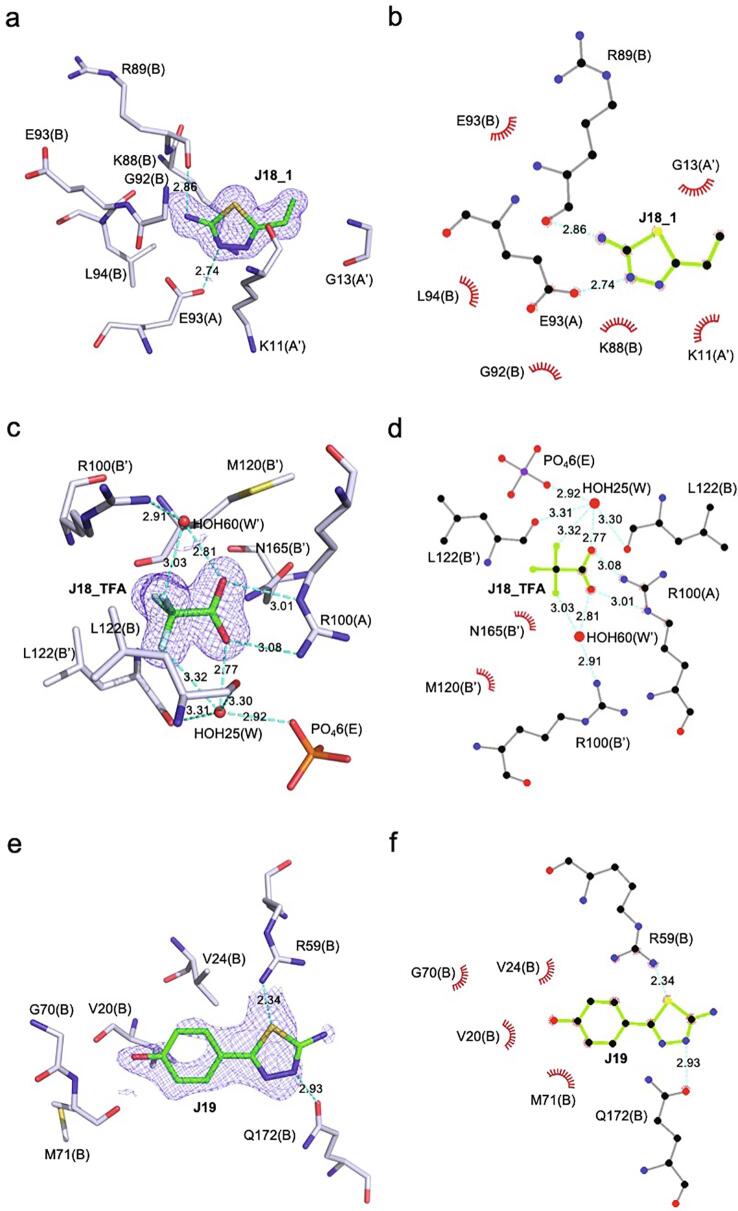
Interactions made by fragments J18 and J19. The amide bond within J18 has apparently been cleaved and the resulting fragments, 2-ethyl-1,3,4-thiadiazole and trifluoroacetic acid, bind at sites D (a, b) and C (c, d), respectively. J19 binds at site E (e, f). These are shown in 3D with the omit electron density contoured at 1.0 RMSD. Hydrogen bonds are indicated by dashed lines in cyan and hydrophobic interactions are indicated by red eyebrow-like icons. Protein chain identifiers are indicated by the letters A and B in brackets and those with a prime are from symmetry-related chains.

Two of the fragments (J18 and J19, Fig. 8) were found to bind at unrelated sites involving crystal contacts which are probably not of physiological significance. Site D lies close to Lys11, Lys88 and Glu93 whereas site E lies between Arg59 and the C-terminal end of the enzyme. The amide bond within J18 has apparently been cleaved and the resulting fragments, trifluoroacetic acid and 2-ethyl-1,3,4-thiadiazole, bind at sites C and D, respectively. Interestingly, it appears that the amide bond in J11 has also been cleaved and the resulting 2-ethyl-1,3,4-thiadiazole binds instead at site C. A check on the stock solution of this compound was made mass spectrometry and this yielded a main mass of 130 Da, which is within a dalton of the predicted molecular mass of the observed fragment. It is possible that the electron withdrawing groups on the amino terminal side of the amide bonds of these two compounds may render them unstable in water.

## 4 Discussion

The X-ray structure of the Southampton virus 3CL^pro^ has been determined at 1.3 Å resolution in a crystal form that has allowed fragment-screening for novel inhibitors to be undertaken at similar resolutions. Two fragments were found to bind in the active site cleft of the protease. J01 and J02 bind in different subsites of the long active site (see Fig. 5) but both of them interact with the functionally important *β*-hairpin linking strands *β*9 and *β*10. J01 occupies S_1_ and forms hydrophobic interactions with catalytic Cys139 while J02 occupies S_2_ and forms hydrophobic and π-π interactions with Glu54 and His30, which are also from the catalytic triad. Both J01 and J02 could potentially be developed into more potent norovirus protease inhibitors, however, a better ligand might ultimately be obtained by coupling them together, given that the distance between the closest two atoms is slightly less than 3.8 Å.

Some of the remaining fragments were found to interact with the protease at its putative RNA-binding site. Whilst these compounds are likely to have less effect on the protease activity than J01 and J02, which bind in the active site, RNA binding to the enzyme has been shown to cause non-competitive inhibition of the protease (Viswanathan et al., 2013). Other fragments were found to bind at an additional site which is buried deeply in the centre of the crystallographic tetramer. The fact that a C193A mutant of the Minerva virus protease forms the same tetramer in the crystal with the C-terminus of one subunit occupying the active site cleft of another monomer (Muzzarelli et al., 2019), suggests that this assembly may also be involved in proteolytic maturation of noroviruses. Hence, compounds that have the potential to interfere with formation of the tetramer or affect its stability may impact on noroviral replication and therefore deserve to be screened for *in vivo* activity, e.g. against mouse norvirus, which can be cultured, or in a suitable replicon assay. If such studies were to be successful, the highly symmetric nature of the binding site is something that could, in principle, be exploited in drug design.

Given the recent COVID-19 pandemic, it is potentially useful to compare our results on SV3CP with the 3CL^pro^ of coronavirus (e.g. Yang et al., 2013). The two enzymes have quite low sequence identity of approximately 12% within the common protease moieties and superimpose with an RMSD of 2.4 Å for 126 structurally aligned residues. The coronavirus protease is considerably larger (303 residues) than SV3CP due to the presence of a C-terminal domain which is involved in dimerisation. Although topologically similar, the protease moieties of both structures differ very substantially in the loop regions connecting the core β-strands. In spite of these differences, coronavirus protease also has specificity for Gln at the P_1_ position of substrate. In very recent fragment screening of the SARS-CoV-2 protease, 23 active site hits were obtained which span the S_3_ to S_1_′ subsites of the enzyme, thus providing somewhat better coverage of the active site cleft than we have achieved with SV3CP (Douangamath et al., 2020). Other SARS-CoV-2 protease inhibitor structures have also been reported in recent months (Dai et al., 2020, Jin et al., 2020a, Jin et al., 2020b, Zhang et al., 2020). This resurgence of interest in rational 3CL^pro^ drug design is likely to have combined benefits for what are currently intractable and severe viral infections. These studies provide a rational basis on which compounds with improved potency can be designed by medicinal chemists.

## CRediT authorship contribution statement

**Jingxu Guo:** Conceptualization, Investigation, Methodology, Writing - original draft. **Alice Douangamath:** Methodology. **Weixiao Song:** Methodology, Investigation. **Alun R. Coker:** Supervision, Investigation. **A.W. Edith Chan:** Supervision, Investigation. **Steve P. Wood:** Supervision, Investigation. **Jonathan B. Cooper:** Supervision, Investigation, Writing - review & editing. **Efrat Resnick:** Investigation, Methodology. **Nir London:** Conceptualization, Methodology, Supervision. **Frank von Delft:** Conceptualization, Methodology, Supervision.

## Declaration of Competing Interest

The authors declare that they have no known competing financial interests or personal relationships that could have appeared to influence the work reported in this paper.

## References

[b0005] Adams P.D., Afonine P.V., Bunkoczi G., Chen V.B., Davis I.W., Echols N., Headd J.J., Hung L.W., Kapral G.J., Grosse-Kunstleve R.W., McCoy A.J., Moriarty N.W., Oeffner R., Read R.J., Richardson D.C., Richardson J.S., Terwilliger T.C., Zwart P.H. (2010). PHENIX: a comprehensive Python-based system for macromolecular structure solution. Acta Crystallogr. Sect. D Biol. Crystallogr..

[b0010] Afonine P.V., Grosse-Kunstleve R.W., Echols N., Headd J.J., Moriarty N.W., Mustyakimov M., Terwilliger T.C., Urzhumtsev A., Zwart P.H., Adams P.D. (2012). Towards automated crystallographic structure refinement with phenix.refine. Acta Crystallogr. Sect. D Biol. Crystallogr..

[b0015] Anand K., Palm G.J., Mesters J.R., Siddell S.G., Ziebuhr J., Hilgenfeld R. (2002). Structure of coronavirus main proteinase reveals combination of a chymotrypsin fold with an extra α-helical domain. EMBO J..

[b0020] Andino R., Rieckhof G.E., Baltimore D. (1990). A functional ribonucleoprotein complex forms around the 5′ end of poliovirus RNA. Cell.

[b0025] Baert L., Uyttendaele M., Stals A., Van Coillie E., Dierick K., Debevere J., Botteldoorn N. (2009). Reported foodborne outbreaks due to noroviruses in Belgium: the link between food and patient investigations in an international context. Epidemiol. Infect..

[b0030] Bartsch S.M., Lopman B.A., Ozawa S., Hall A.J., Lee B.Y. (2016). Global Economic Burden of Norovirus Gastroenteritis. PLoS One.

[b0035] Bauman J.D., Patel D., Baker S.F., Vijayan R.S.K., Xiang A., Parhi A.K., Martínez-Sobrido L., LaVoie E.J., Das K., Arnold E. (2013). Crystallographic Fragment Screening and Structure-Based Optimization Yields a New Class of Influenza Endonuclease Inhibitors. ACS Chem. Biol..

[b0040] Bazan J.F., Fletterick R.J. (1988). Viral cysteine proteases are homologous to the trypsin-like family of serine proteases: structural and functional implications. PNAS.

[b0045] Bergmann E.M., Mosimann S.C., Chernaia M.M., Malcolm B.A., James M.N. (1997). The Refined Crystal Structure of the 3C Gene Product From Hepatitis A Virus: Specific Proteinase Activity and RNA Recognition. J. Virol..

[b0050] Bernstein D.I., Atmar R.L., Lyon G.M., Treanor J.J., Chen W.H., Jiang X., Vinjé J., Gregoricus N., Frenck R.W., Moe C.L. (2015). Norovirus vaccine against experimental human GII. 4 virus illness: a challenge study in healthy adults. J. Infect. Dis..

[b0055] Birtley J.R., Knox S.R., Jaulent A.M., Brick P., Leatherbarrow R.J., Curry S. (2005). Crystal structure of foot-and-mouth disease virus 3C protease new insights into catalytic mechanism and cleavage specificity. J. Biol. Chem..

[b0060] Blakeney S.J., Cahill A., Reilly P.A. (2003). Processing of Norwalk virus nonstructural proteins by a 3C-like cysteine proteinase. Virology.

[b0065] Boniotti B., Wirblich C., Sibilia M., Meyers G., Thiel H.-J., Rossi C. (1994). Identification and characterization of a 3C-like protease from rabbit hemorrhagic disease virus, a calicivirus. J. Virol..

[b0070] Chang K.O., Takahashi D., Prakash O., Kim Y. (2012). Characterization and inhibition of norovirus proteases of genogroups I and II using a fluorescence resonance energy transfer assay. Virology.

[b0075] ChemAxon, L. 2013. Marvin Sketch 6.0.0.

[b0080] Chen V.B., Arendall W.B., Headd J.J., Keedy D.A., Immormino R.M., Kapral G.J., Murray L.W., Richardson J.S., Richardson D.C. (2010). MolProbity: all-atom structure validation for macromolecular crystallography. Acta Crystallogr. Sect. D Biol. Crystallogr..

[b0085] Clarke I., Estes M., Green K., Hansman G., Knowles N., Koopmans M., Matson D., Meyers G., Neill J., Radford A. (2012). Caliciviridae. Virus Taxonomy.

[b0090] Clarke I.N., Lambden P.R. (1997). The molecular biology of caliciviruses. J. General Virol..

[b0095] Collins P.M., Ng J.T., Talon R., Nekrosiute K., Krojer T., Douangamath A., Brandao-Neto J., Wright N., Pearce N.M., von Delft F. (2017). Gentle, fast and effective crystal soaking by acoustic dispensing. Acta Crystallogr. Sect. D: Struct. Biol..

[b0100] Cox O.B., Krojer T., Collins P., Monteiro O., Talon R., Bradley A., Fedorov O., Amin J., Marsden B.D., Spencer J. (2016). A poised fragment library enables rapid synthetic expansion yielding the first reported inhibitors of PHIP (2), an atypical bromodomain. Chem. Sci..

[b0105] Dai W., Zhang B., Jiang X.-M., Su H., Li J., Zhao Y., Xie X., Jin Z., Peng J., Liu F., Li C., Li Y., Bai F., Wang H., Cheng X.i., Cen X., Hu S., Yang X., Wang J., Liu X., Xiao G., Jiang H., Rao Z., Zhang L.-K., Xu Y., Yang H., Liu H. (2020). Structure-based design of antiviral drug candidates targeting the SARS-CoV-2 main protease. Science.

[b0110] Damalanka V.C., Kim Y., Kankanamalage A.C.G., Lushington G.H., Mehzabeen N., Battaile K.P., Lovell S., Chang K.-O., Groutas W.C. (2017). Design, synthesis, and evaluation of a novel series of macrocyclic inhibitors of norovirus 3CL protease. Eur. J. Med. Chem..

[b0115] Delbart F., Brams M., Gruss F., Noppen S., Peigneur S., Boland S., Chaltin P., Brandao-Neto J., von Delft F., Touw W.G., Joosten R.P., Liekens S., Tytgat J., Ulens C. (2018). An allosteric binding site of the α7 nicotinic acetylcholine receptor revealed in a humanized acetylcholine-binding protein. J. Biol. Chem..

[b0120] Division of Viral Diseases, N.C.f.I., Respiratory Diseases, C.f.D.C., Prevention, 2011. Updated norovirus outbreak management and disease prevention guidelines. MMWR. Recommendations and reports: Morbidity and mortality weekly report. Recommendations and reports 60, 1–18. 21368741

[b0125] Douangamath A., Fearon D., Gehrtz P., Krojer T., Lukacik P., Owen C.D., Resnick E., Strain-Damerell C., Aimon A., Ábrányi-Balogh P., Brandaõ-Neto J., Carbery A., Davison G., Dias A., Downes T.D., Dunnett L., Fairhead M., Firth J.D., Jones S.P., Keely A., Keserü G.M., Klein H.F., Martin M.P., Noble M.E.M., O’Brien P., Powell A., Reddi R., Skyner R., Snee M., Waring M.J., Wild C., London N., von Delft F., Walsh M.A. (2020). Crystallographic and electrophilic fragment screening of the SARS-CoV-2 main protease. BioRxiv.

[b0130] Emmott E., de Rougemont A., Hosmillo M., Lu J., Fitzmaurice T., Haas J., Goodfellow I. (2019). Polyprotein processing and intermolecular interactions within the viral replication complex spatially and temporally control norovirus protease activity. J. Biol. Chem..

[b0135] Emsley P., Cowtan K. (2004). Coot: model-building tools for molecular graphics. Acta Crystallogr. Sect. D Biol. Crystallogr..

[b0140] Fernandes H., Leen E.N., Cromwell H., Pfeil M.-P., Curry S. (2015). Structure determination of Murine Norovirus NS6 proteases with C-terminal extensions designed to probe protease-substrate interactions. PeerJ.

[b0145] Florescu D.F., Hill L.A., McCartan M.A., Grant W. (2008). Two cases of Norwalk virus enteritis following small bowel transplantation treated with oral human serum immunoglobulin. Pediatr. Transplant..

[b0150] Hussey R.J., Coates L., Gill R.S., Erskine P.T., Coker S.F., Mitchell E., Cooper J.B., Wood S., Broadbridge R., Clarke I.N., Lambden P.R., Shoolingin-Jordan P.M. (2011). A structural study of norovirus 3C protease specificity: binding of a designed active site-directed peptide inhibitor. Biochemistry.

[b0155] Incardona M.F., Bourenkov G.P., Levik K., Pieritz R.A., Popov A.N., Svensson O. (2009). EDNA: a framework for plugin-based applications applied to X-ray experiment online data analysis. J. Synchrotron Radiat..

[b0160] Jiang X., Wang M., Wang K., Estes M.K. (1993). Sequence and genomic organization of Norwalk virus. Virology.

[b0165] Jin Zhenming, Du Xiaoyu, Xu Yechun, Deng Yongqiang, Liu Meiqin, Zhao Yao, Zhang Bing, Li Xiaofeng, Zhang Leike, Peng Chao, Duan Yinkai, Yu Jing, Wang Lin, Yang Kailin, Liu Fengjiang, Jiang Rendi, Yang Xinglou, You Tian, Liu Xiaoce, Yang Xiuna, Bai Fang, Liu Hong, Liu Xiang, Guddat Luke W., Xu Wenqing, Xiao Gengfu, Qin Chengfeng, Shi Zhengli, Jiang Hualiang, Rao Zihe, Yang Haitao (2020). Structure of Mpro from SARS-CoV-2 and discovery of its inhibitors. Nature.

[b0170] Jin Zhenming, Zhao Yao, Sun Yuan, Zhang Bing, Wang Haofeng, Wu Yan, Zhu Yan, Zhu Chen, Hu Tianyu, Du Xiaoyu, Duan Yinkai, Yu Jing, Yang Xiaobao, Yang Xiuna, Yang Kailin, Liu Xiang, Guddat Luke W., Xiao Gengfu, Zhang Leike, Yang Haitao, Rao Zihe (2020). Structural basis for the inhibition of SARS-CoV-2 main protease by antineoplastic drug carmofur. Nat. Struct. Mol. Biol..

[b0175] Kankanamalage A.C.G., Kim Y., Weerawarna P.M., Uy R.A.Z., Damalanka V.C., Mandadapu S.R., Alliston K.R., Mehzabeen N., Battaile K.P., Lovell S. (2015). Structure-guided design and optimization of dipeptidyl inhibitors of norovirus 3CL protease. Structure-activity relationships and biochemical, X-ray crystallographic, cell-based, and in vivo studies. J. Med. Chem..

[b0180] Kantardjieff K.A., Rupp B. (2003). Matthews coefficient probabilities: Improved estimates for unit cell contents of proteins, DNA, and protein-nucleic acid complex crystals. Protein Sci.: Publ. Protein Soc..

[b0185] Kawatkar S., Gagnon M., Hoesch V., Tiong-Yip C., Johnson K., Ek M., Nilsson E., Lister T., Olsson L., Patel J. (2016). Design and Structure-Activity Relationships of Novel Inhibitors of Human Rhinovirus 3C Protease. Bioorg. Med. Chem. Lett..

[b0190] Kim Y., Lovell S., Tiew K.-C., Mandadapu S.R., Alliston K.R., Battaile K.P., Groutas W.C., Chang K.-O. (2012). Broad-spectrum antivirals against 3C or 3C-like proteases of picornaviruses, noroviruses, and coronaviruses. J. Virol..

[b0195] Krissinel E., Henrick K. (2007). Inference of macromolecular assemblies from crystalline state. J. Mol. Biol..

[b0200] Krojer T., Talon R., Pearce N., Collins P., Douangamath A., Brandao-Neto J., Dias A., Marsden B., von Delft F. (2017). The XChemExplorer graphical workflow tool for routine or large-scale protein–ligand structure determination. Acta Crystallogr. Sect. D: Struct. Biol..

[b0205] Lambden P.R., Caul E.O., Ashley C.R., Clarke I.N. (1993). Sequence and genome organization of a human small round-structured (Norwalk-like) virus. Science.

[b0210] Leen E.N., Baeza G., Curry S. (2012). Structure of a murine norovirus NS6 protease-product complex revealed by adventitious crystallisation. PLoS One.

[b0215] Leong L.E., Walker P.A., Porter A.G. (1993). Human rhinovirus-14 protease 3C (3Cpro) binds specifically to the 5'-noncoding region of the viral RNA. Evidence that 3Cpro has different domains for the RNA binding and proteolytic activities. J. Biol. Chem..

[b0220] Lew J.F., Kapikian A.Z., Valdesuso J., Green K.Y. (1994). Molecular characterization of Hawaii virus and other Norwalk-like viruses: evidence for genetic polymorphism among human caliciviruses. J. Infect. Dis..

[b0225] Lew J.F., Kapikian A.Z., Jiang X., Estes M.K., Green K.Y. (1994). Molecular characterization and expression of the capsid protein of a Norwalk-like virus recovered from a Desert Shield troop with gastroenteritis. Virology.

[b0230] Liu L., Johnson H.L., Cousens S., Perin J., Scott S., Lawn J., Rudan I., Campbell H., Cibulskis R., Li M. (2012). Child Health Epidemiology Reference Group of WHO and UNICEF Global, regional, and national causes of child mortality: an updated systematic analysis for 2010 with time trends since 2000. Lancet.

[b0235] Lochridge V.P., Hardy M.E. (2003). Snow Mountain virus genome sequence and virus-like particle assembly. Virus Genes.

[b0240] Lucero Y., Vidal R., O'Ryan G.M. (2018). Norovirus vaccines under development. Vaccine.

[b0245] Mandadapu S.R., Weerawarna P.M., Gunnam M.R., Alliston K.R., Lushington G.H., Kim Y., Chang K.-O., Groutas W.C. (2012). Potent inhibition of norovirus 3CL protease by peptidyl α-ketoamides and α-ketoheterocycles. Bioorg. Med. Chem. Lett..

[b0250] Mateo R., Lindesmith L.C., Garg S.J., Gottlieb K., Lin K., Said S., Leon J.S., Sims A.C., Weber D.J., Baric R.S., Tucker S.N., Taylor D.N. (2020). Production and Clinical Evaluation of Norwalk GI.1 Virus Lot 001–09NV in Norovirus Vaccine Development. J. Infect. Dis..

[b0255] McCoy A.J., Grosse-Kunstleve R.W., Adams P.D., Winn M.D., Storoni L.C., Read R.J. (2007). Phaser crystallographic software. J. Appl. Crystallogr..

[b0260] McFadden N., Bailey D., Carrara G., Benson A., Chaudhry Y., Shortland A., Heeney J., Yarovinsky F., Simmonds P., Macdonald A., Goodfellow I. (2011). Norovirus regulation of the innate immune response and apoptosis occurs via the product of the alternative open reading frame 4. PLoS Pathog..

[b0265] Mosimann S.C., Cherney M.M., Sia S., Plotch S., James M.N. (1997). Refined X-ray crystallographic structure of the poliovirus 3C gene product. J. Mol. Biol..

[b0270] Murshudov G.N., Skubak P., Lebedev A.A., Pannu N.S., Steiner R.A., Nicholls R.A., Winn M.D., Long F., Vagin A.A. (2011). REFMAC5 for the refinement of macromolecular crystal structures. Acta Crystallogr. Sect. D Biol. Crystallogr..

[b0275] Muzzarelli K.M., Kuiper B., Spellmon N., Brunzelle J., Hackett J., Amblard F., Zhou S., Liu P., Kovari I.A., Yang Z., Schinazi R.F., Kovari L.C. (2019). Structural and Antiviral Studies of the Human Norovirus GII.4 Protease. Biochem..

[b0280] Nakamura K., Someya Y., Kumasaka T., Ueno G., Yamamoto M., Sato T., Takeda N., Miyamura T., Tanaka N. (2005). A norovirus protease structure provides insights into active and substrate binding site integrity. J. Virol..

[b0285] Nayak A., Goodfellow I.G., Woolaway K.E., Birtley J., Curry S., Belsham G.J. (2006). Role of RNA Structure and RNA Binding Activity of Foot-and-Mouth Disease Virus 3C Protein in VPg Uridylylation and Virus Replication. J. Virol..

[b0290] Netzler N.E., Enosi Tuipulotu D., White P.A. (2019). Norovirus antivirals: Where are we now?. Med. Res. Rev..

[b0295] Ng J.T., Dekker C., Kroemer M., Osborne M., von Delft F. (2014). Using textons to rank crystallization droplets by the likely presence of crystals. Acta Crystallogr. D Biol. Crystallogr..

[b0300] Pearce N., Bradley A.R., Collins P., Krojer T., Nowak R., Talon R., Marsden B.D., Kelm S., Shi J., Deane C. (2016). A Multi-Crystal Method for Extracting Obscured Signal from. Crystallogr. Electr. Density..

[b0305] Phillips G., Tam C.C., Conti S., Rodrigues L.C., Brown D., Iturriza-Gomara M., Gray J., Lopman B. (2010). Community incidence of norovirus-associated infectious intestinal disease in England: improved estimates using viral load for norovirus diagnosis. Am. J. Epidemiol..

[b0310] Resnick E., Bradley A., Gan J., Douangamath A., Krojer T., Sethi R., Geurink P.P., Aimon A., Amitai G., Bellini D., Bennett J., Fairhead M., Fedorov O., Gabizon R., Gan J., Guo J., Plotnikov A., Reznik N., Ruda G.F., Díaz-Sáez L., Straub V.M., Szommer T., Velupillai S., Zaidman D., Zhang Y., Coker A.R., Dowson C.G., Barr H.M., Wang C., Huber K.V.M., Brennan P.E., Ovaa H., von Delft F., London N. (2019). Rapid Covalent-Probe Discovery by Electrophile-Fragment Screening. J. Am. Chem. Soc..

[b0315] Scallan E., Hoekstra R.M., Angulo F.J., Tauxe R.V., Widdowson M.A., Roy S.L., Jones J.L., Griffin P.M. (2011). Foodborne illness acquired in the United States–major pathogens. Emerg. Infect. Dis..

[b0320] Siebenga J.J., Vennema H., Zheng D.-P., Vinjé J., Lee B.E., Pang X.-L., Ho E.C., Lim W., Choudekar A., Broor S. (2009). Norovirus illness is a global problem: emergence and spread of norovirus GII. 4 variants, 2001–2007. J. Infect. Dis..

[b0325] Someya Y., Takeda N., Miyamura T. (2002). Identification of active-site amino acid residues in the Chiba virus 3C-like protease. J. Virol..

[b0330] Terwilliger T.C., Grosse-Kunstleve R.W., Afonine P.V., Moriarty N.W., Adams P.D., Read R.J., Zwart P.H., Hung L.-W. (2008). Iterative-build OMIT maps: map improvement by iterative model building and refinement without model bias. Acta Crystallogr. D Biol. Crystallogr..

[b0335] Thorne L.G., Goodfellow I.G. (2014). Norovirus gene expression and replication. J. Gen. Virol..

[b0340] Tiew K.-C., He G., Aravapalli S., Mandadapu S.R., Gunnam M.R., Alliston K.R., Lushington G.H., Kim Y., Chang K.-O., Groutas W.C. (2011). Design, synthesis, and evaluation of inhibitors of Norwalk virus 3C protease. Bioorg. Med. Chem. Lett..

[b0345] Vega E., Barclay L., Gregoricus N., Shirley S.H., Lee D., Vinje J. (2014). Genotypic and epidemiologic trends of norovirus outbreaks in the United States, 2009 to 2013. J. Clin. Microbiol..

[b0350] Vinjé J. (2015). Advances in laboratory methods for detection and typing of norovirus. J. Clin. Microbiol..

[b0355] Viskovska M.A., Zhao B., Shanker S., Choi J.-M., Deng L., Song Y., Palzkill T., Hu L., Estes M.K., Venkataram P.B.V. (2019). Norovirus Protease Shows pH-Sensitive Proteolysis with a Unique Arg-His Pairing in the Catalytic Site. J. Virol..

[b0360] Viswanathan P., May J., Uhm S., Yon C., Korba B. (2013). RNA binding by human Norovirus 3C-like proteases inhibits protease activity. Virology.

[b0365] Wallace A.C., Laskowski R.A., Thornton J.M. (1995). LIGPLOT: a program to generate schematic diagrams of protein-ligand interactions. Protein Eng..

[b0370] Winter G. (2010). xia2: an expert system for macromolecular crystallography data reduction. J. Appl. Crystallogr..

[b0375] Wojdyr M., Keegan R., Winter G., Ashton A. (2013). DIMPLE - a pipeline for the rapid generation of difference maps from protein crystals with putatively bound ligands. Acta Crystallogr. Sect. A.

[b0380] Yang H., Yang M., Ding Y., Liu Y., Lou Z., Zhou Z., Sun L., Mo L., Ye S., Pang H., Gao G.F., Anand K., Bartlam M., Hilgenfeld R., Rao Z. (2013). The crystal structures of severe acute respiratory syndrome virus main protease and its complex with an inhibitor. PNAS.

[b0385] Zeitler C.E., Estes M.K., Prasad B.V. (2006). X-ray crystallographic structure of the Norwalk virus protease at 1.5-Å resolution. J. Virol..

[b0390] Zhang L., Lin D., Sun X., Curth U., Drosten C., Sauerhering L., Becker S., Rox K., Hilgenfeld R. (2020). Crystal structure of SARS-CoV-2 main protease provides a basis for design of improved α-ketoamide inhibitors. Science.

[b0395] Zhang R., McIntyre P.J., Collins P.M., Foley D.J., Arter C., von Delft F., Bayliss R., Warriner S., Nelson A. (2019). Construction of a Shape-Diverse Fragment Set: Design, Synthesis and Screen against Aurora-A Kinase. Chemistry.

[b0400] Zwart P., Grosse-Kunstleve R., Adams P. (2005). Xtriage and Fest: automatic assessment of X-ray data and substructure structure factor estimation. CCP4 Newsl.

