# Supplementary material for: In crystallo-screening for discovery of human norovirus 3C-like protease inhibitors
Source: J Struct Biol X. 2020 Jul 16;4:100031. doi: 10.1016/j.yjsbx.2020.100031 (PMC7365090; doi:10.1016/j.yjsbx.2020.100031)

**Supplementary Material**

**Supplementary Fig. 1.** A superposition of the tetramers found in the crystal structures of the 3CL protease from Southampton virus (red), Chiba virus (green), Houston virus (yellow), Minerva virus (cyan) and mouse norovirus (blue). The corresponding RCSB ID’s are 2iph, 1wqs, 6nir, 6b6i and 4x2v, respectively.


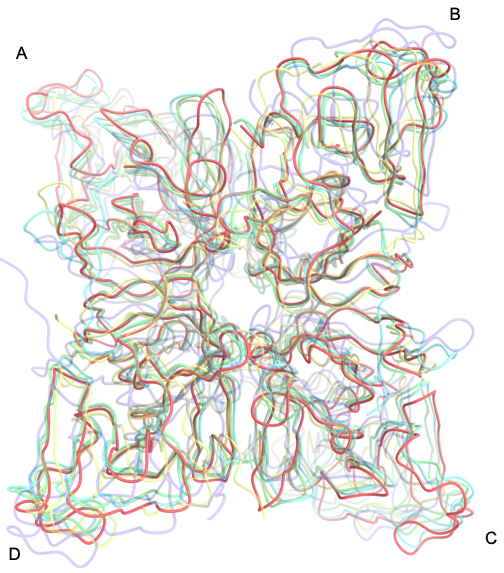


**Supplementary Fig. 2.** Mass-spectrum of the native SV3CL protease.


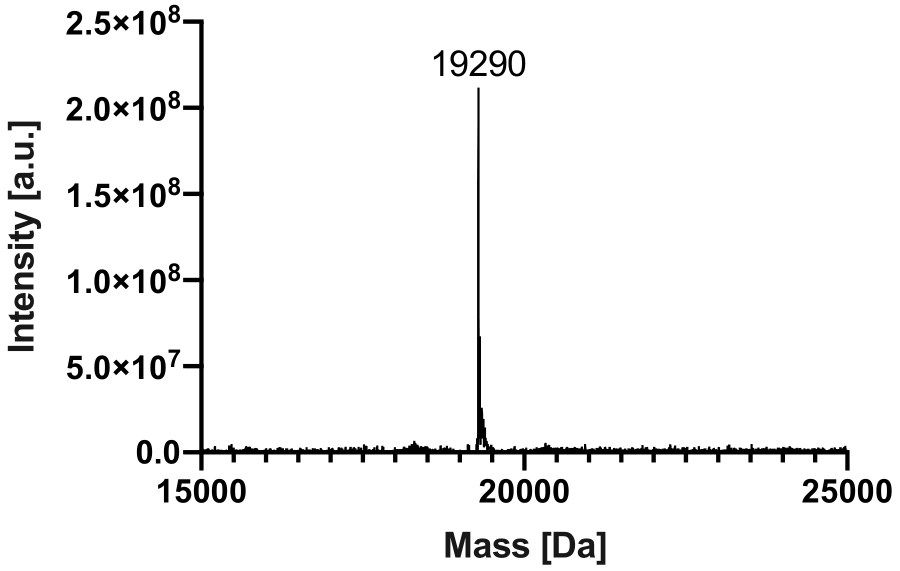

Supplement: Supplementary data 1 [file mmc1.docx]
